# Human brain sialoglycan ligand for CD33, a microglial inhibitory Siglec implicated in Alzheimer’s disease

**DOI:** 10.1016/j.jbc.2022.101960

**Published:** 2022-04-20

**Authors:** Anabel Gonzalez-Gil, Ryan N. Porell, Steve M. Fernandes, Eila Maenpaa, T. August Li, Tong Li, Philip C. Wong, Kazuhiro Aoki, Michael Tiemeyer, Zaikuan J. Yu, Benjamin C. Orsburn, Namandjé N. Bumpus, Russell T. Matthews, Ronald L. Schnaar

**Affiliations:** 1 Department of Pharmacology and Molecular Sciences, Johns Hopkins University School of Medicine, Baltimore, Maryland, USA; 2 Department of Pathology, Johns Hopkins University School of Medicine, Baltimore, Maryland, USA; 3 Department of Neuroscience, Johns Hopkins University School of Medicine, Baltimore, Maryland, USA; 4 Complex Carbohydrate Research Center, University of Georgia, Athens, Georgia, USA; 5 Department of Neuroscience and Physiology, State University of New York Upstate Medical University, Syracuse, New York, USA

**Keywords:** Alzheimer's disease, microglia, sialic acid, Siglec-3, Siglec-8, Siglec-F, sialyltransferase, receptor protein tyrosine phosphatase zeta, phosphacan, keratan sulfate, AD, Alzheimer's disease, GuHCl, guanidinium hydrochloride, IgG, immunoglobulin G, LC, liquid chromatography, mCD33, mouse CD33, MS, mass spectrometry, MWCO, molecular weight cutoff, NIH, National Institutes of Health, PBST, Dulbecco's PBS supplemented with 0.1% Tween-20, PVDF, polyvinylidene fluoride, RPTPζ, receptor protein tyrosine phosphatase zeta, Siglec, sialic acid–binding immunoglobulin-type lectin

## Abstract

Alzheimer’s disease (AD) is characterized by accumulation of misfolded proteins. Genetic studies implicate microglia, brain-resident phagocytic immune cells, in AD pathogenesis. As positive effectors, microglia clear toxic proteins, whereas as negative effectors, they release proinflammatory mediators. An imbalance of these functions contributes to AD progression. Polymorphisms of human CD33, an inhibitory microglial receptor, are linked to AD susceptibility; higher CD33 expression correlates with increased AD risk. CD33, also called Siglec-3, is a member of the sialic acid–binding immunoglobulin-type lectin (Siglec) family of immune regulatory receptors. Siglec-mediated inhibition is initiated by binding to complementary sialoglycan ligands in the tissue environment. Here, we identify a single sialoglycoprotein in human cerebral cortex that binds CD33 as well as Siglec-8, the most abundant Siglec on human microglia. The ligand, which we term receptor protein tyrosine phosphatase zeta (RPTPζ)^S3L^, is composed of sialylated keratan sulfate chains carried on a minor isoform/glycoform of RPTPζ (phosphacan) and is found in the extracellular milieu of the human brain parenchyma. Brains from human AD donors had twofold higher levels of RPTPζ^S3L^ than age-matched control donors, raising the possibility that RPTPζ^S3L^ overexpression limits misfolded protein clearance contributing to AD pathology. Mice express the same structure, a sialylated keratan sulfate RPTPζ isoform, that binds mouse Siglec-F and crossreacts with human CD33 and Siglec-8. Brains from mice engineered to lack RPTPζ, the sialyltransferase *St3gal4*, or the keratan sulfate sulfotransferase *Chst1* lacked Siglec binding, establishing the ligand structure. The unique CD33 and Siglec-8 ligand, RPTPζ^S3L^, may contribute to AD progression.

Alzheimer’s disease (AD) is characterized by accumulation of misfolded proteins, amyloid β extracellularly and phosphorylated tau intracellularly (1). Genome-wide association studies of AD susceptibility identified several genes expressed predominantly by microglia, the resident immune cells of the brain that are involved in debris clearance and neuroinflammation (2, 3, 4, 5). Among microglial genes consistently associated with AD susceptibility is *CD33* (6, 7, 8), also known as Siglec-3, a member of the sialic acid–binding immunoglobulin-type lectin (Siglec) family of immune regulatory cell surface transmembrane receptors (9, 10). Most Siglecs are expressed on the surface of immune cells, and most, including CD33, downregulate immune responses of cells on which they are expressed. Each Siglec binds to sialic acid–containing glycans carried on glycoproteins or glycolipids in their local environment (11). Siglec binding to its complementary ligands initiates signaling to modulate the activity of the cells on which they are expressed, including microglia (12, 13, 14, 15).

Increased CD33 expression associates with increased AD susceptibility, whereas expression of CD33 lacking a sialic acid–binding domain reduces risk (3, 15), implicating CD33-mediated limitation of amyloid clearance as the mechanism for enhanced AD susceptibility. *In vitro* expression of human CD33 in microglia inhibits Aβ42 clearance, whereas the splice variant lacking sialic acid binding does not (15). These data imply that binding of microglial CD33 to its endogenous sialoglycan ligands in the brain inhibits phagocytosis, reduces clearance of misfolded proteins, and contributes to AD progression. If this is the case, knowledge of the structures of CD33 ligands in the brain, the sialoglycan ligands that bind to CD33 on microglia, will provide insight into this disease-modifying signaling pathway. We describe such a complementary binding sialoglycan ligand in human (and mouse) brain.

In this report, we identify and characterize a single ∼1 MDa sialoglycoprotein ligand expressed in human cerebral cortex that binds both human CD33 and Siglec-8, the most abundant inhibitory Siglec expressed by human microglia. A ligand with nearly identical molecular and binding properties was found in mouse brain as a ligand for Siglec-F, a mouse microglial inhibitory Siglec. Expression of the CD33 ligand is increased in the cerebral cortex of human AD tissue donors compared with age-matched nondemented donors.

## Results

### CD33 binds to a single sialoglycoprotein from human cerebral cortex

**Figure 1:**
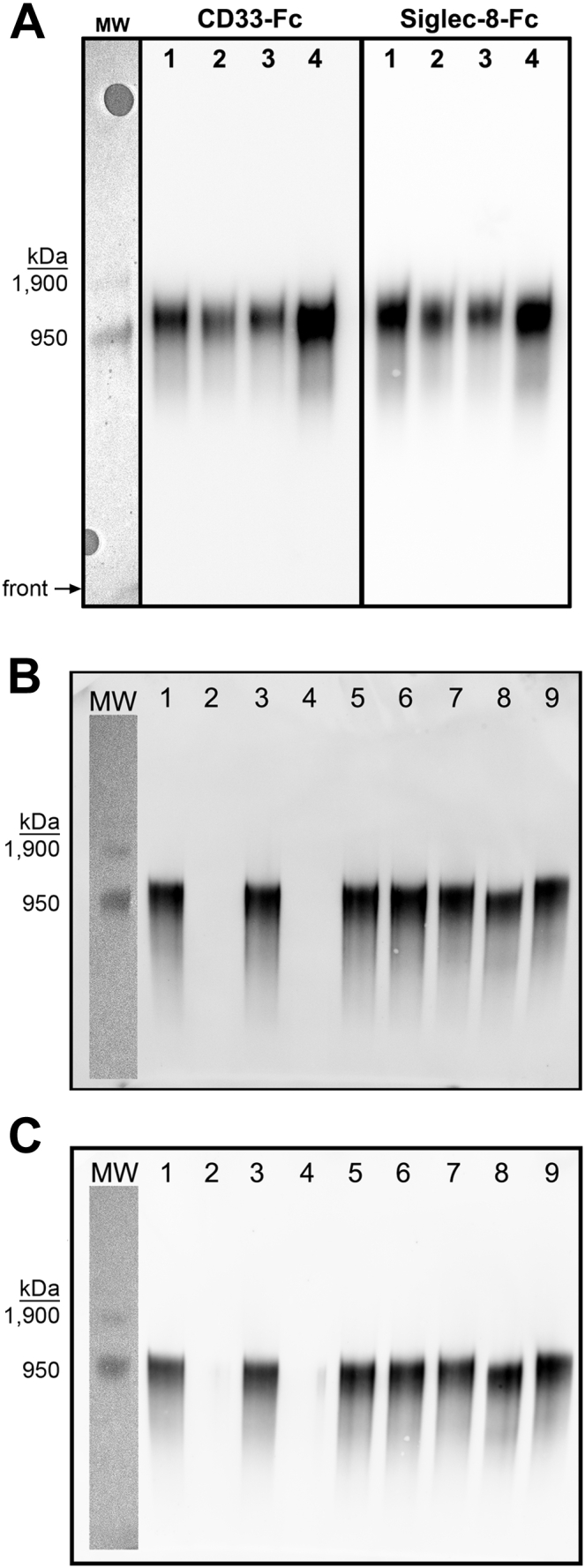
**Extraction of human cerebral cortex proteins and resolution by gel electrophoresis reveals a single species that binds CD33 (Siglec-3) and Siglec-8.***A*, total protein extracts from human cortex (inferior parietal lobe) from four different tissue donors (AD brains, males, age range 65–76 years) were resolved on composite agarose–acrylamide gels, blotted to PVDF, probed for Siglec ligands using human CD33-Fc and Siglec-8-Fc, and detected by enhanced chemiluminescence. Sample lanes were flanked by prestained crosslinked IgM (950 kDa major band, 1.9 MDa minor band) detected by white light. The entire length of the gel blot is presented, with the *front* denoted by an *arrow*. *B* and *C*, cerebral cortex extract from a single AD donor was dialyzed against sodium phosphate buffer and incubated under matched control conditions (without enzyme) or with enzymes prior to resolution on composite agarose–acrylamide gels, blotting to PVDF, and probing with CD33-Fc (*B*) or Siglec-8-Fc (*C*). Sample lanes were flanked by prestained crosslinked IgM detected by white light. Lanes are as follows: (1) sialidase control buffer; (2) 120 mU/ml sialidase; (3) keratanase I control buffer; (4) 8.4 mU/ml keratanase I; (5) keratanase II control buffer; (6) 8.4 mU/ml keratanase II; (7) PNGase F control buffer; (8) PNGase F; and (9) no treatment or incubation. AD, Alzheimer’s disease; IgM, immunoglobulin M; PVDF, polyvinylidene fluoride; Siglec, sialic acid–binding immunoglobulin-type lectin.

To search for ligands for CD33 in human brain, guanidinium hydrochloride (GuHCl) buffer was used to thoroughly extract glycoproteins from human cerebral cortical tissue from four individual donors. Extracted proteins were resolved by composite agarose–acrylamide gel electrophoresis to separate large glycoproteins, blotted to a polyvinylidene fluoride (PVDF) membrane, and overlaid with soluble CD33-Fc chimera to detect CD33-binding ligands. Remarkably, among all the sialoglycans in the human brain, a single large (∼1 MDa) protein bound CD33-Fc in extracts from each of the four donors (Fig. 1*A*).

**Figure 2:**
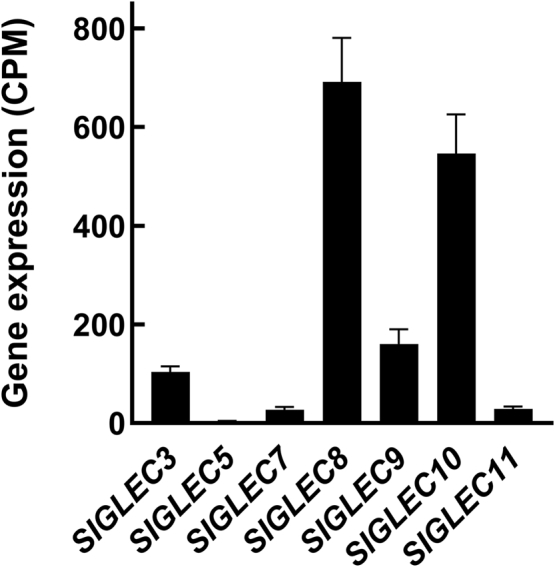
**Microglial expression of inhibitory Siglecs.** Microglia isolated from postmortem control (nondemented) human cerebral cortexes were subjected to bulk transcriptomic analysis. Data were mined from Supporting Information provided by Alsema *et al.* (17) and are presented as mean ± SEM (n = 10). CPM, counts per million reads mapped; Siglec, sialic acid–binding immunoglobulin-type lectin.

Human microglia express not only CD33 but also other inhibitory Siglecs (16, 17), with SIGLEC8 > SIGLEC10 > SIGLEC9 ≈ CD33 >> SIGLEC7 ≈ SIGLEC11 (Fig. 2). To search for ligands for these other inhibitory human microglial Siglecs, the same extracts were screened with their Siglec-Fc chimeras. Notably, Siglec-8-Fc bound robustly to a single ligand with the same migration and donor-to-donor variation as CD33-Fc (Fig. 1*A*).

Based on binding to synthetic glycan arrays and NMR-binding isotherms (18, 19, 20, 21), Siglec-8 is highly selective, requiring a sialic acid in α2–3 linkage to a galactose that carries a sulfate ester on its 6-carbon hydroxyl, for example, Neu5Acα2–3[6-SO_4_]Galβ1–4GlcNAc, for optimal binding. CD33 binds most avidly to this same glycan (21). In other tissues, this structure is carried on terminally sialylated keratan sulfate chains (22, 23). To test whether the human brain CD33 and Siglec-8 ligands were likewise sialylated keratan sulfates, brain protein extract was treated with glycohydrolases (Fig. 1, *B* and *C*). Binding of CD33-Fc and Siglec-8-Fc was completely abrogated by pretreatment with sialidase or keratanase I. Keratanase II, which cleaves keratan sulfate chains in highly sulfated stretches, was without effect. PNGase F, which cleaves N-linked (but not O-linked) glycans from glycoproteins resulted in a shift in migration but retention of CD33 and Siglec-8 binding. Together, these data predict that CD33 and Siglec-8 ligand from human brain are large proteoglycans that carry O-linked terminally sialylated keratan sulfate chains.

### CD33 and Siglec-8 ligand in human brain is carried on receptor protein tyrosine phosphatase zeta

**Figure 3:**
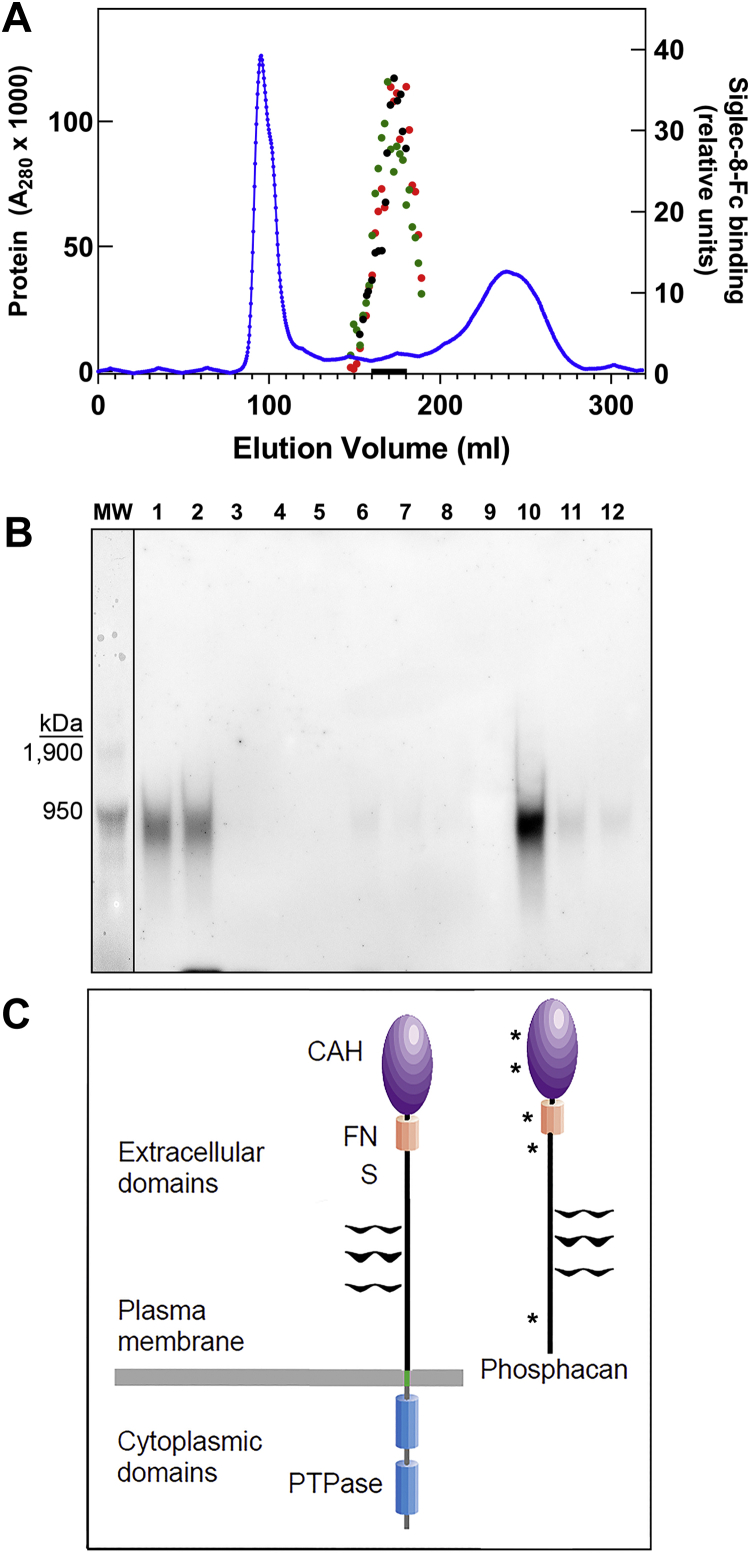
**Purification of human brain Siglec-8 ligand by size-exclusion chromatography and affinity capture.***A*, human cerebral cortex extract was resolved by Sephacryl S-500 size-exclusion chromatography. Protein elution was followed at 280 nm (*blue line*), fractions were collected for electrophoretic resolution, blotting, and probing with Siglec-8-Fc detected with HRP-antihuman Fc, and quantified by ECL image analysis (*black circles*). Fractions containing Siglec-8 ligand were combined for further purification as indicated by the *black bar* on the *X*-axis. In a separate size-exclusion fractionation on the same column, aliquots of fractions were resolved by electrophoresis in duplicate, blotted, and probed separately with Siglec-8-Fc (*green circles*) and CD33-Fc (*red circles*). *B*, combined size-exclusion fractions (*black bar*, panel *A*) were incubated with Siglec-8-Fc–adsorbed Protein G magnetic beads, washed, and ligand eluted with increased salt concentration. Fractions were resolved on composite agarose–acrylamide gels, blotted, probed with Siglec-8-Fc precomplexed to HRP-conjugated antihuman Fc and Siglec bound to ligand detected by ECL. *Lanes*: MW, prestained crosslinked IgM MW standards; major band 950 kDa, minor band 1.9 MDa; (1) precapture; (2) precleared on IgG beads; (3) flow through (unbound) Siglec-8-Fc beads; (4–8) low salt washes; (9) MW marker (not visible by ECL); and (10–12) high salt elutions. *C*, proteomic MS revealed receptor type protein phosphatase zeta (RTPTζ, also known as RPTPβ), which exists as a transmembrane form (*left*) and released extracellular domain (phosphacan, *right*). Both isoforms contain a carbonic anhydrase domain (CAH), a fibronectin type III repeat (FN), and a spacer domain (S). Glycosaminoglycans are shown as *black wavy lines*. The transmembrane forms contain a transmembrane domain (*green*) and cytoplasmic tyrosine phosphatase domains (*PTPase*). All peptides identified in purified Siglec-8 ligand (*asterisks*) are found on both forms. Image modified from Ref. (57). ECL, enhanced chemiluminescence; HRP, horseradish peroxidase; IgM, immunoglobulin M; MW, molecular weight; Siglec, sialic acid–binding immunoglobulin-type lectin.

A three-step procedure was developed to purify the human brain CD33 and Siglec-8 ligand. Brain extract was subjected to differential ethanol precipitation, the resulting proteins resolved by size-exclusion chromatography, and the ligand purified by affinity chromatography. During purification, CD33-Fc and Siglec-8-Fc binding ligands tracked with one another (Fig. 3). Upon Sephacryl S-500 size-exclusion chromatography, designed for large macromolecule separations up to several million daltons, the ligand was well separated from most large and small brain proteins (Fig. 3*A*). Subsequent capture on Siglec-8-Fc affinity beads and elution with high salt buffer (Fig. 3*B*) provided sufficient purification for proteomic mass spectrometry (MS). Multiple MS analyses revealed receptor protein tyrosine phosphatase zeta (RPTPζ) (UniProt: P23471, previously called RPTPβ) as the top proteomic match (Table 1) with five high-confidence peptides detected in two separate MS systems. RPTPζ is a large brain proteoglycan (2315 amino acids) that exists both as a transmembrane protein tyrosine phosphatase and a released extracellular form known as phosphacan (Fig. 3*C*). The high-confidence peptides detected in the purified Siglec ligand span the extracellular domain. Mass spectrometric details for the peptides identified are presented in Table S1.

**Table 1:** RPTPζ peptides identified by proteomic MS in Siglec-8 ligand purified from human brain

Amino acid number	Sequence	Xcorr^a^ and/or Amanda^b^ score
112	VSGGVSEMVFK	3.54^a^/268^b^
195	AIIDGVESVSR	268^b^
346	FAVLYQQLDGEDQTK	4.51^a^
436	DIEEGAIVNPGRD	4.19^a^
1442	CMSCSSYR	185^b^

Peptide positions and sequences for peptides identified with high confidence from purified human brain Siglec-8 ligand are shown. Peptides were matched to receptor-type tyrosine-protein phosphatase zeta (UniProtKB: P23471). Peptides were identified using two analytical systems with the quality of hits reported.

a SEQUEST-HT Xcorr score.

b MS Amanda 2.0 score.

**Figure 4:**
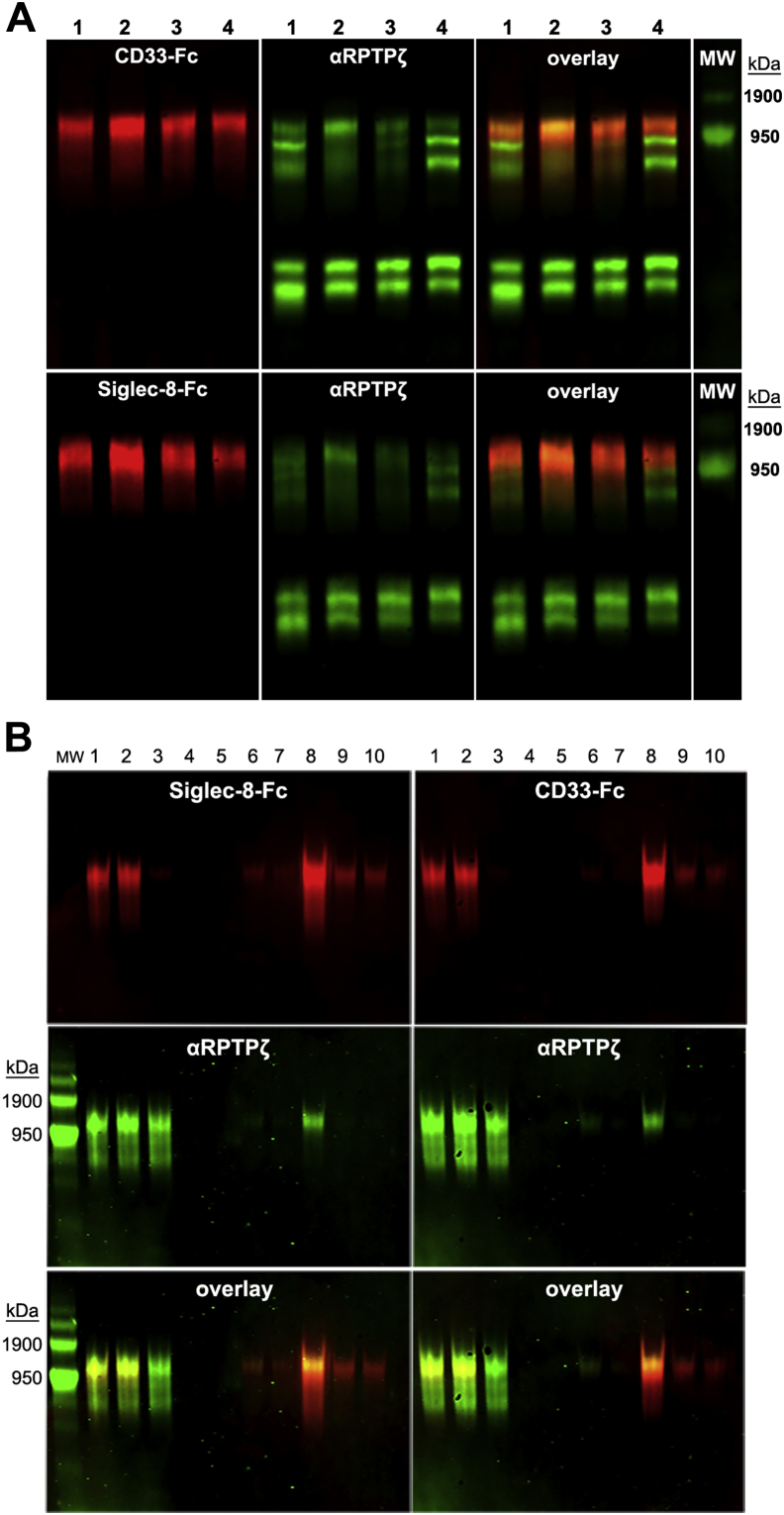
**The same glycoform of RPTPζ carries CD33 and Siglec-8 ligands.***A*, equal aliquots of human cerebral cortex total protein extract from four donors (numbered) were resolved on replicate composite agarose–acrylamide gels and blotted to PVDF. One blot (*upper panels*) was double-label probed with CD33-Fc (*red*) and anti-RPTPζ (*green*) and a replicate blot (*lower panels*) with Siglec-8-Fc (*red*) and anti-RPTPζ (*green*). *B*, human cerebral cortex extract was size excluded (not shown) and subjected to affinity capture purification as for Figure 3. Equal aliquots of samples from affinity capture were resolved on replicate composite agarose–acrylamide gels, blotted to PVDF, and double-label probed with Siglec-8-Fc (*red*) and anti-RPTPζ (*green*) or with CD33-Fc (*red*) and anti-RPTPζ (*green*) as indicated. Lanes: (1) precapture; (2) precleared on IgG beads; (3) flow through (unbound) Siglec-8-Fc beads; (4–7) low salt washes; and (8–10) high salt elutions. The double-label gels carried custom molecular weight markers visible in the *green images* only. IgG, immunoglobulin G; PVDF, polyvinylidene fluoride; RPTPζ, receptor protein tyrosine phosphatase zeta; Siglec, sialic acid–binding immunoglobulin-type lectin.

Validation that the CD33 and Siglec-8 ligand from human brain is a large isoform of RPTPζ was obtained by electrophoretic comigration and copurification (Fig. 4). Electrophoretic resolution of human brain extract from four donors revealed a single ∼1 MDa human CD33-Fc/Siglec-8-Fc binding component and five isoforms of RPTPζ, three large and two small (small isoforms of RPTPζ do not carry glycosaminoglycan chains (24)). The largest isoform migrated with CD33-Fc and Siglec-8-Fc binding (Fig. 4*A*).

After size-exclusion chromatography, only the large isoforms remained (Fig. 4*B*, lanes 1–2). Affinity purification on Siglec-8-Fc beads captured all the CD33 and Siglec-8 ligand, which coeluted (lane 8). A portion of the largest isoform of RPTPζ coeluted with the CD33/Siglec-8 ligand. Notably, much of the large isoform of RPTPζ did not bind to the Siglec-8-Fc beads (lane 3) and did not bind Siglec-8-Fc or CD33-Fc, indicating that the CD33/Siglec-8 ligand constitutes a subpopulation—a specific glycoform—of the largest RPTPζ molecular weight isoform. We conclude that a portion of the large isoform of RPTPζ is post-translationally modified to carry sialylated keratan sulfate chains that bind both CD33 and Siglec-8, whereas most RPTPζ isoforms and glycoforms fail to bind these Siglecs. For simplicity, we refer to this glycoform/isoform as RPTPζ^S3L^ (Siglec-3 ligand) to emphasize that the ligand is not RPTPζ *per se* but a specific minor isoform and glycoform.

When purified RPTPζ^S3L^ was treated with keratanase I or sialidase as for Figure 1, Siglec binding was totally abrogated, whereas anti-RPTPζ immunoblotting was retained (Fig. S1). These data further demonstrate that RPTPζ protein *per se* does not bind Siglecs without its key sialoglycan keratan sulfate chain elaborations.

### Expression of Siglec ligand is increased in the brains of donors with AD

**Figure 5:**
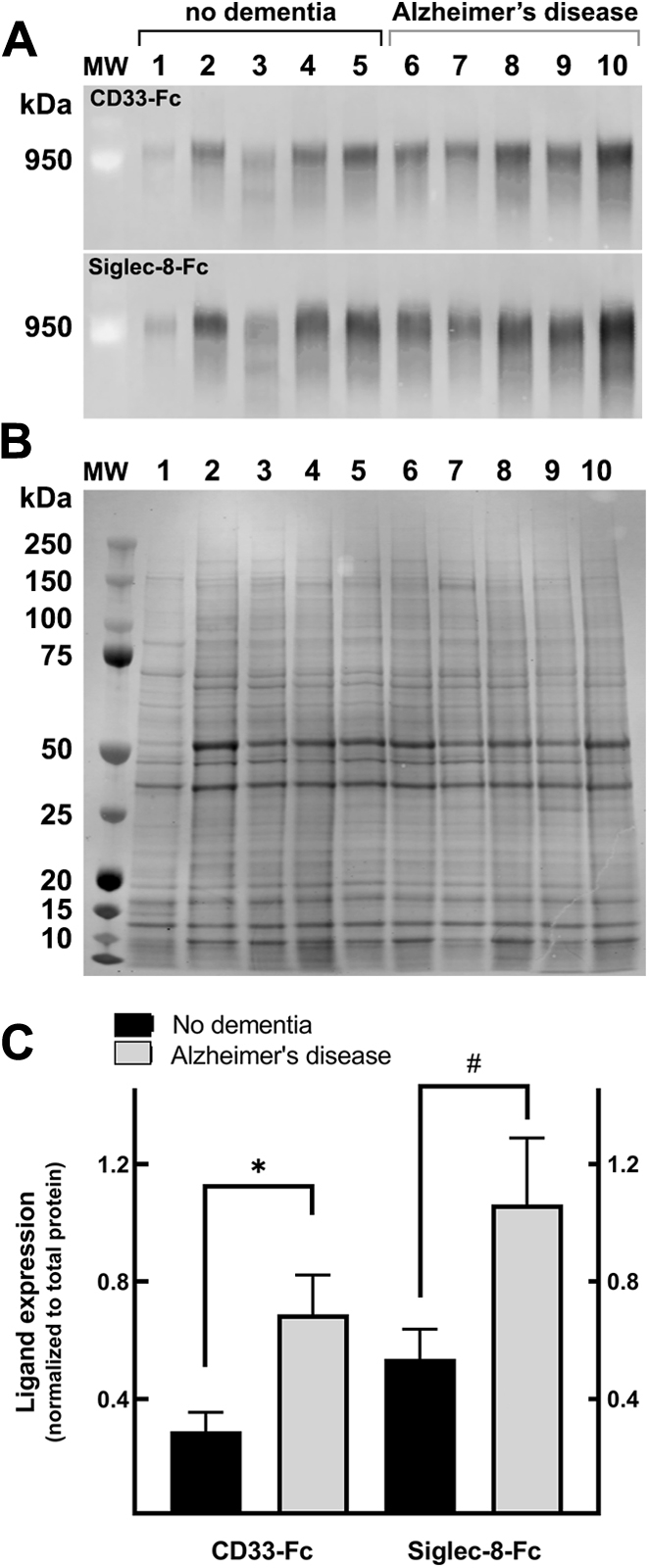
**Human CD33 and Siglec-8 ligand expression in control (nondemented) and AD cerebral cortex.** Proteins were extracted and resolved on replicate composite agarose–acrylamide gels (*A*) to resolve large proteins. PVDF blots were probed with CD33-Fc or Siglec-8-Fc precomplexed with goat antihuman IgG, Fc specific. Binding was detected using IRDye 800CW conjugated donkey antigoat IgG and near-infrared fluorescent imaging (LI-COR). A custom molecular weight marker of prelabeled crosslinked IgM is shown. For normalization of the Siglec-Fc blots, equal aliquots from each donor were resolved and on a 4 to 12% acrylamide gel to resolve total extracted proteins (*B*). PVDF blots were then stained with LI-COR Revert 700 total protein stain. Bio-Rad Precision Plus standards are shown. Lanes 1 to 5, control donor samples; lanes 6 to 10, AD donor samples. *C*, quantification of CD33-Fc and Siglec-8-Fc band densities normalized to total protein (LI-COR Image Studio). ∗*p* = 0.028; ^#^*p* = 0.067. AD, Alzheimer’s disease; IgG, immunoglobulin G; IgM, immunoglobulin M; PVDF, polyvinylidene fluoride; Siglec, sialic acid–binding immunoglobulin-type lectin.

Inferior parietal cortex samples, gyrus cross-sectional blocks with mixed gray and white matter, were obtained from five AD donors (average age, 74.4 years) and five age-matched nondemented control donors (average age, 76.6 years; Table 2). Proteins from each tissue sample were extracted under identical conditions, and equal volume of aliquots were resolved on replicate composite agarose–acrylamide gels and blotted to PVDF membranes. Membranes were subjected to near-infrared fluorescent double labeling using anti-RPTPζ and CD33-Fc or anti-RPTPζ and Siglec-8-Fc. Siglec ligands detected by CD33-Fc or Siglec-8-Fc overlay binding are shown in Figure 5*A* and accompanying anti-RPTPζ immunooverlay in Fig. S2. Separate aliquots were resolved on 4 to 12% acrylamide gels for total protein quantification used to normalize the Siglec overlay and anti-RPTPζ immuno-overlay data (Fig. 5*B*).

**Table 2:** Human tissue donors

Donor code^a^	Age	Dementia	Braak	Sex
1	68	None	—	M
2	87	None	2	F
3	79	None	1	M
4	76	None	—	M
5	73	None	—	M
6	89	AD	6	F
7	73	AD	6	M
8	69	AD	6	M
9	76	AD	6	M
10	65	AD	6	M

Abbreviations: F, female; M, male.

a Electrophoresis lane in Figure 5.

The density of CD33-Fc and Siglec-8-Fc binding normalized to total protein (Fig. 5*C*) is increased in AD donor samples compared with age-matched controls (2.4-fold for CD33-Fc, *p* = 0.028; 2.0-fold for Siglec-8-Fc, *p* = 0.067). In contrast, immunoblot staining intensity ratios of total RPTPζ isoforms between AD donor samples and nondemented controls was statistically unchanged, and staining intensity of the largest RPTPζ isoform was likewise unchanged (Fig. S2).

### Humans and mice express different Siglecs but the same brain Siglec ligand

**Figure 6:**
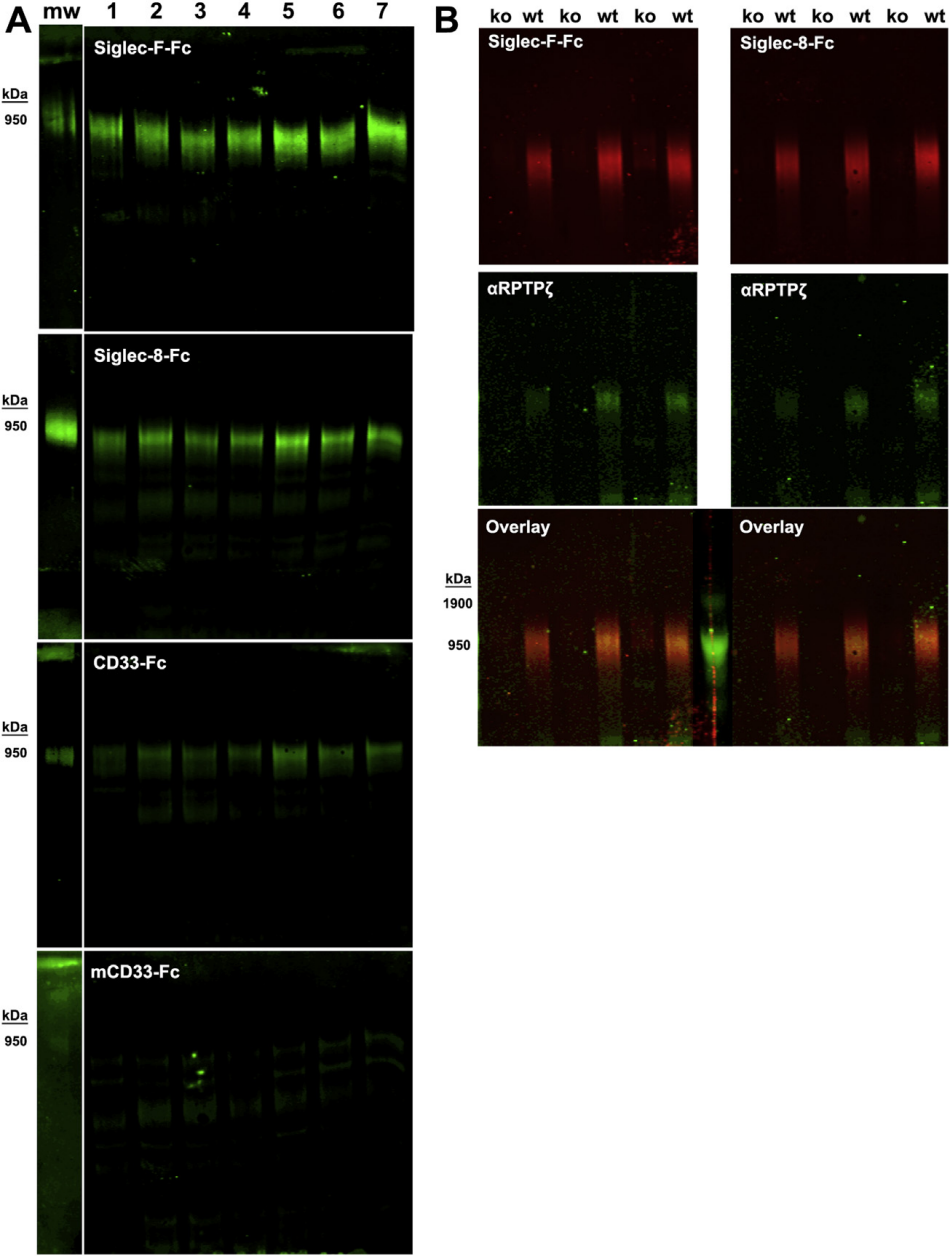
**Mouse brain expresses Siglec-F ligand on RPTPζ that binds Siglec-8 and CD33 but not mCD33.***A*, extracts from human cerebral cortex (lane 1) and six mouse brains (lanes 2–7) were resolved on composite agarose–acrylamide gels, blotted to PVDF, and probed for Siglec ligands using the indicated Siglec-Fc chimeras precomplexed with goat antihuman IgG. Binding was detected using IRDye 800CW-conjugated donkey antigoat IgG followed by near-infrared fluorescent imaging (LI-COR). Lanes 2 to 4 were from Tau transgenic mice and lanes 5 to 7 from wildtype mice (58). *B*, mouse Siglec-F ligand comigrates with mouse RPTPζ and is lost in *Ptprz*1-null mouse brain. Brains from three wildtype and three *Ptprz1*-null littermates were extracted, and aliquots were resolved in duplicate on a composite agarose–acrylamide gel, transferred to PVDF, then the membrane cut into replicate sets of lanes. One set was probed with Siglec-F-Fc and the other with Siglec-8-Fc, each precomplexed to IRDye 800CW antihuman IgG (*red*). Blots were double labeled with anti-RPTPζ detected with IRDye 680RD anti-rabbit IgG (*green*). Custom prestained molecular weight markers were centrally loaded and appear *green* on the overlay blots. The two overlay blots were separately probed and recombined prior to imaging with the recombined molecular weight markers centrally located. IgG, immunoglobulin G; mCD33, mouse CD33; PVDF, polyvinylidene fluoride; RPTPζ, receptor protein tyrosine phosphatase zeta; Siglec, sialic acid–binding immunoglobulin-type lectin.

Human Siglecs diverged extensively from those in mice, such that several mouse Siglecs are designated by letters rather than numbers (9, 10, 25). Importantly, mouse CD33 (mCD33) is structurally and functionally different than its human counterpart; inhibition of phagocytosis by human CD33 is not conserved in mCD33 (13). For this reason, we refer to the human protein as CD33 in this report and the mouse protein as mCD33. Mouse microglia express inhibitory Siglecs-F, -G, and -H (26). An unbiased phosphoproteome screen of multiple mouse AD models revealed that Siglec-F, a paralog of human Siglec-8, is uniquely upregulated on reactive microglia (27). When we screened mouse brain extracts with mouse Siglecs, Siglec-F-Fc alone bound reproducibly and robustly to a single protein (∼1 MDa) that comigrated with purified human RPTPζ^S3L^ (Fig. 6).

Mouse brains were extracted, the proteins resolved by composite agarose–acrylamide gels, blotted to PVDF, and overlaid with different mouse and human Siglec-Fc chimeras (Fig. 6*A*). Among mouse Siglecs tested, only Siglec-F-Fc displayed robust binding primarily to a single glycoprotein that migrated at the same position as the human CD33/Siglec-8 ligand. mCD33 failed to bind robustly to any species. Blot overlay with Fc chimeras of mouse Siglec-E, Siglec-G, and Siglec-H was weak and did not bind to the Siglec-F ligand (data not shown). Human CD33-Fc and Siglec-8-Fc bound to mouse extracts at the same migration position as Siglec-F-Fc (Fig. 6*A*).

**Figure 7:**
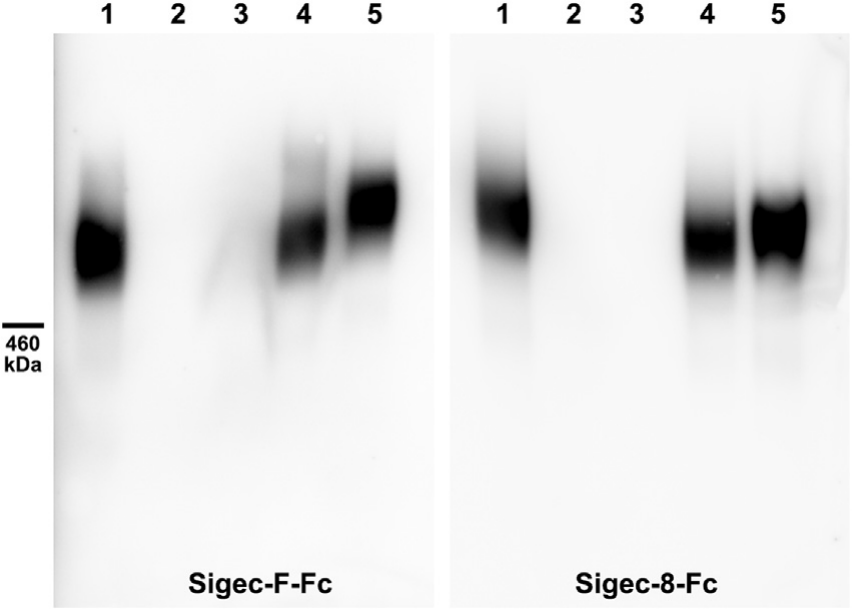
**Mouse brain Siglec-F ligand is a sialylated keratan sulfate.** Mouse brain extract was pretreated with buffer alone or with buffer containing glycosidases. Samples were resolved by composite agarose–acrylamide gel electrophoresis, blotted to PVDF, and blots were probed with Siglec-F-Fc or Siglec-8-Fc precomplexed with HRP-conjugated antihuman Fc. Siglec binding was detected by enhanced chemiluminescence. Lanes: (1) control; (2) 2 U/ml sialidase; (3) 8.4 mU/ml keratanase I; (4) 75 mU/ml keratanase II; and (5) 0.5 U/ml chondroitinase ABC. HRP, horseradish peroxidase; PVDF, polyvinylidene fluoride; Siglec, sialic acid–binding immunoglobulin-type lectin.

**Figure 8:**
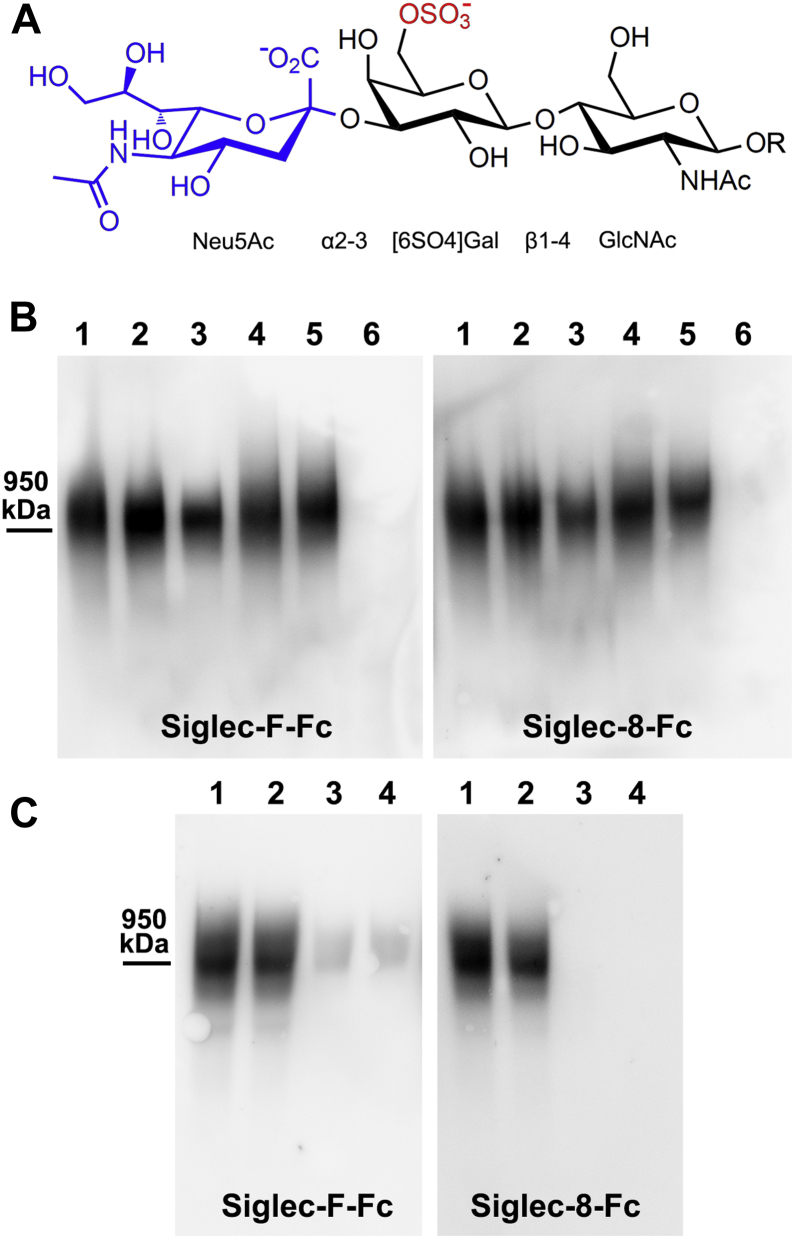
**Disruption of *St3gal4* or *Chst1* genes diminishes Siglec-F ligand expression in mouse brain.***A*, minimal common structure for binding Siglec-F and Siglec-8 (19, 20). Key components include the α2–3-linked sialic acid (*blue*) and the sulfate ester on the 6-carbon hydroxyl of galactose (*red*). *B*, brains of mice genetically engineered with disrupted α2–3 sialyltransferases were extracted, resolved on composite agarose–acrylamide gels, proteins blotted to PVDF, and probed with Siglec-F-Fc or Siglec-8-Fc precomplexed with HRP-conjugated antihuman Fc. Binding was detected by enhanced chemiluminescence. Equivalent protein loading was confirmed by 4 to 12% polyacrylamide gel electrophoresis and total protein staining (not shown). Lanes: (1) wildtype; (2) *St3gal1*-null; (3) *St3gal2*-null; (4) *St3gal3*-null; (5) *St3gal2/3*-double-null; and (6) *St3gal4*-null. *C*, brain proteins from mice genetically engineered with disrupted carbohydrate sulfotransferase (*Chst1*) gene were extracted, resolved, and probed as for panel *B*. Lanes: (1 and 2) wildtype; (3 and 4) *Chst1*-null. HRP, horseradish peroxidase; PVDF, polyvinylidene fluoride; Siglec, sialic acid–binding immunoglobulin-type lectin.

To test whether mouse brain Siglec-F ligand is carried on RPTPζ, Siglec-F-Fc binding was compared in wildtype and *Ptprz1*-null mice, which do not express RPTPζ. Consistent with RPTPζ being the sole carrier of Siglec-F ligand in the mouse brain, Siglec-F-Fc and anti-RPTPζ staining comigrated, and both were absent in *Ptprz1*-null mice (Fig. 6*B*). To test whether the mouse brain Siglec ligand shares glycosylation properties with the human ligand, mouse brain extract was treated with glycohydrolases (Fig. 7). Consistent with the properties of the human Siglec ligand, binding of Siglec-F-Fc (as well as Siglec-8-Fc) to the mouse brain ligand was abrogated by pretreatment with sialidase and keratanase I but not by keratanase II or chondroitinase ABC. These data support the conclusion that human and mouse brain express a similar sialylated keratan sulfate exclusively carried on RPTPζ that engages inhibitory microglial Siglecs. We refer to this mouse ligand and RPTPζ^SFL^ (Siglec-F ligand). Notably, glycan array binding (20) supports the conclusion that CD33, Siglec-8, and Siglec-F bind robustly to the sulfated sialylated trisaccharide Neu5Acα2–3[6SO4]Galβ1–4GlcNAc (Fig. 8*A*).

To identify biosynthetic genes responsible for the brain Siglec ligand in mice, brains from genetically engineered mice were used. There are six α2–3 sialyltransferase genes in mice (and humans), of which four were tested (Fig. 8*B*). Extracted proteins from the brains of mice lacking *St3gal4* were devoid of Siglec-F-Fc binding, whereas binding to proteins from mice with disrupted *St3gal1*, *St3gal2*, or *St3gal3* genes remained robust. Likewise, brain extracts from mice with the disrupted *Chst1* gene had greatly diminished or absent Siglec-F-Fc binding (Fig. 8*C*). *Chst1* encodes the enzyme keratan sulfate Gal-6 sulfotransferase, consistent with the finding of keratanase I sensitivity of the Siglec ligand (Fig. 7). Brain extracts from *St3gal4*-null and *Chst1*-null mice also failed to bind Siglec-8-Fc (Fig. 8, *B* and *C*), consistent with the conclusion that the same glycan target is responsible for mouse and human Siglec binding.

**Figure 9:**
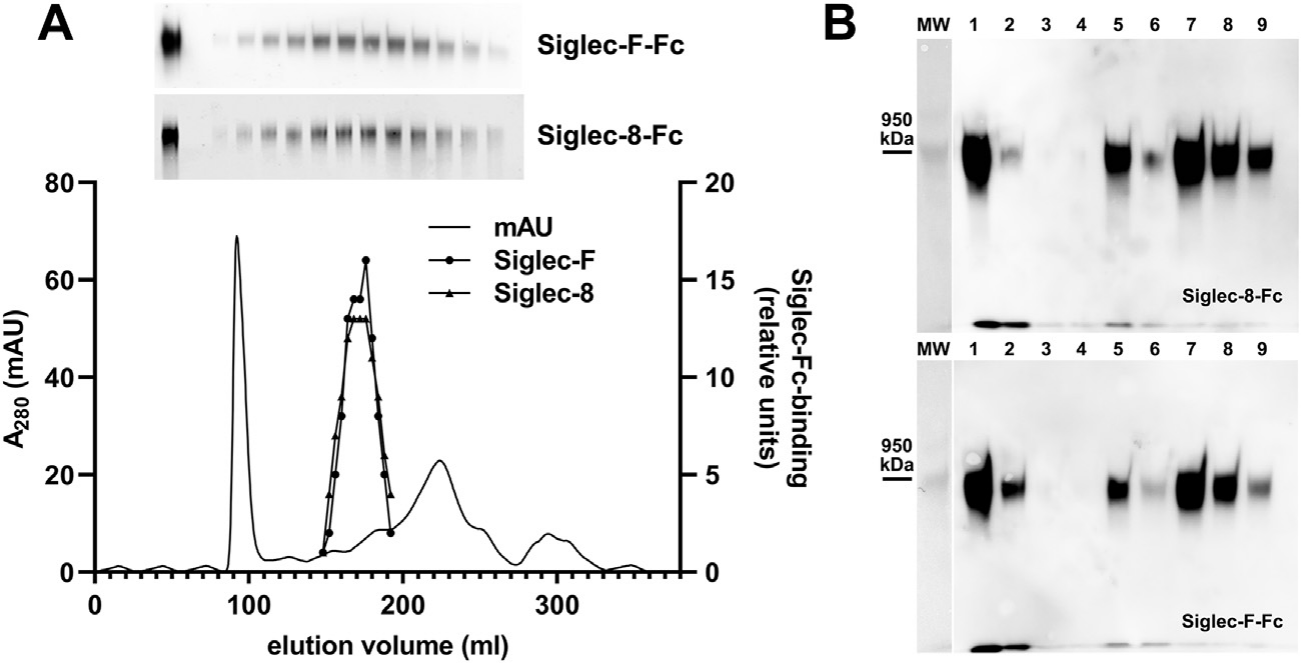
**Mouse brain Siglec-F ligand coelutes with Siglec-8 binding and is purified by Siglec-8-Fc affinity capture.***A*, mouse brain extract was resolved by Sephacryl S-500 size-exclusion chromatography, and fractions were collected for electrophoretic resolution, blotting, and probing with Siglec-F-Fc and Siglec-8-Fc. The *inset* shows the Siglec-Fc overlay blots with input (unfractionated) sample in the *left-most* lane followed by resolved aliquots of even numbered fractions through the peak (fractions 30–54). The *graph* indicates protein (absorbance at 280 nm) and densitometry of the peak fractions (as indicated) as a function of elution volume. Only the peak fractions (shown) contained significant Siglec-binding material. *B*, combined size-exclusion fractions were incubated with Siglec-8-Fc–adsorbed Protein G magnetic beads, washed, and ligand eluted with increased salt. Fractions were resolved on replicate composite agarose–acrylamide gels, blotted, and probed with Siglec-8-Fc and Siglec-F-Fc precomplexed to HRP-conjugated antihuman Fc. Siglecs bound to ligand were detected by enhanced chemiluminescence. Lanes: MW, prestained crosslinked IgM MW standards; major band 950 kDa; (1) sample precleared on IgG beads; (2) flow through (unbound) Siglec-8-Fc beads; (3 and 4) loading buffer washes; (5 and 6) low salt washes; (7–9) and high salt elutions. HRP, horseradish peroxidase; IgG, immunoglobulin G; IgM, immunoglobulin M; MW, molecular weight; Siglec, sialic acid–binding immunoglobulin-type lectin.

Further evidence of similarity between the mouse Siglec-F and human Siglec-8 brain ligands was obtained by copurification of the mouse brain Siglec-F ligand using size-exclusion and Siglec-8-Fc affinity capture. Elution of Siglec-F-Fc and Siglec-8-Fc binding activity *via* size exclusion was identical (Fig. 9*A*), and Siglec-8-Fc efficiently captured mouse Siglec-F ligand (Fig. 9*B*).

### The human shared Siglec ligand—RPTPζ^S3L^—is found extracellularly in the parenchyma of the human cerebral cortex

**Figure 10:**
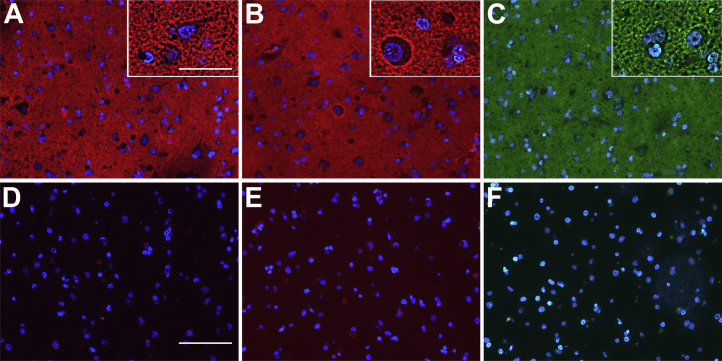
**Human brain CD33/Siglec-8 ligand is distributed throughout the cerebral cortex parenchyma.** Human cerebral cortex tissue sections were overlaid with CD33-Fc (*A*) or Siglec-8-Fc (*B*) precomplexed with goat antihuman Fc or with rabbit anti-RPTPζ antibody (*C*). After washing, Siglec binding was detected with Alexa 594-conjugated donkey antigoat IgG (*red*) and anti-RPTPζ with Alexa 488 donkey anti-rabbit IgG (*green*). Controls included pretreatment of tissue sections with sialidase followed by incubation with precomplexed CD33-Fc (*D*) or Siglec-8-Fc (*E*) or treatment with Alexa 488 donkey anti-rabbit IgG without primary anti-RPTPζ antibody (*F*). *Insets* were computationally sharpened postimage capture at a setting of 400% at 2.6 μm radius. The size bar represents main panels, 100 μm; *insets*, 37 μm. IgG, immunoglobulin G; RPTPζ, receptor protein tyrosine phosphatase zeta; Siglec, sialic acid–binding immunoglobulin-type lectin.

RPTPζ is expressed at cell surfaces as a transmembrane form and in the brain extracellular matrix as the released proteoglycan phosphacan (Fig. 3*C*). To determine the histological distribution of the RPTPζ^S3L^ glycoform of RPTPζ, Siglec-Fc overlay histochemistry was compared with anti-RPTPζ immunohistochemistry (Fig. 10). CD33-Fc, Siglec-8-Fc, and anti-RPTPζ stained in an extracellular reticular pattern in the normal human cerebral cortex. Computational image sharpening revealed some larger cells surrounded by more intense staining (Fig. 10, *insets*). Staining by Siglec-Fc chimeras was lost after pretreatment with sialidase, indicating selective staining to sialoglycans.

## Discussion

Genome-wide association studies reveal that several genetic loci associated with late-onset AD susceptibility are selectively expressed by microglia, implicating microglia as modifiers of AD progression (28). For the immune inhibitory microglial cell surface protein CD33 (Siglec-3), alleles associated with increased expression result in increased AD risk, whereas alleles that truncate the receptor result in decreased risk (3). We infer that the inhibitory activity of CD33, and by extension of other inhibitory Siglecs on human microglia, may limit phagocytosis of misfolded proteins, contributing to disease progression. Binding of human CD33 (and other inhibitory Siglecs) to endogenous complementary sialoglycan ligands mediates microglial inhibition (14, 29). Inhibitory Siglec ligands in the brain, therefore, are potential AD-modifying agents and therapeutic targets.

Human CD33 (human Siglec-3) is not the most abundant inhibitory Siglec expressed by human microglia (Fig. 2), where Siglec-8 and Siglec-10 dominate quantitatively (16, 17). Although Siglec-2 (CD22) was linked to microglial function in aging mice (30, 31), its expression was not reported in human microglia (17, 32). Siglec-1, which is thought to mediate some types of phagocytosis (33), was the only Siglec whose gene expression was different (reduced) in AD microglia compared with no dementia microglia. In an initial screen of the inhibitory Siglecs expressed by human microglia to extracts of normal human cerebral cortices, Siglec-10 and Siglec-11 failed to bind any protein, whereas Siglec-3, Siglec-8, Siglec-7, and Siglec-9 bound to glycoproteins of similar size (Fig. S3*A*). Siglec-3 and Siglec-8 bind to the same entity as shown by crossclearance (Fig. 4*B*), whereas much of the ligand bound by Siglec-7 and Siglec-9 was unbound by Siglec-8-Fc affinity capture (Fig. S3*B*). The identities of those additional Siglec ligands were not investigated further.

A major finding of the current studies is that a single sialoglycoprotein, a quantitatively minor isoform and glycoform of RPTPζ, carries all the CD33 and Siglec-8 binding detected in human cerebral cortex extracts. While we cannot rule out the presence of other ligands that are undetected by our protocols, GuHCl is a thorough method of protein solubilization, and only small alternative ligands (*e.g.*, gangliosides, (21)) may have been missed. The presence of a single large protein carrier of CD33 and Siglec-8 ligands in the human brain is remarkable, in that glycosylation is performed by a suite of biosynthetic enzymes in the Golgi apparatus that presumably encounter many other proteins on their way from the endoplasmic reticulum to the cell surface (34). Nevertheless, the brain sialoglycan ligand for CD33 and Siglec-8 is expressed on a single protein, RPTPζ. How this specification occurs is a compelling biosynthetic question. The evolutionary choice of RPTPζ as the carrier of Siglec ligands in the brain is conserved from mouse to human. This finding was also notable, in that the Siglec family of immune regulatory proteins has undergone significant evolutionary changes over mammalian evolution (10, 35). mCD33 is structurally and functionally distinct from its human counterpart, and nine other human Siglecs do not have mouse homologs, including Siglec-8. Nevertheless, mice express a brain Siglec-F sialoglycan ligand carried exclusively on RPTPζ that crossreacts with human CD33 and Siglec-8. Notably, knockout of RPTPζ in mice (Fig. 6*B*) did not result in alternative biosynthesis of the Siglec glycan ligand on any other protein. This finding emphasizes the high protein specificity of the glycosylation machinery involved and implies that the RPTPζ polypeptide backbone engages the biosynthetic machinery in the Golgi complex to direct the biosynthesis of this evolutionarily conserved Siglec ligand.

In a screen of multiple Siglec-Fc constructs binding to brain extracts, only CD33 and Siglec-8 bound exclusively to the RPTPζ isoform, whereas Siglec-6, Siglec-9, Siglec-10, and Siglec-11 did not (Fig. S3 and data not shown). Among mouse Siglec-Fc constructs tested, only Siglec-F consistently bound the RPTPζ isoform, whereas mCD33, Siglec-E, Siglec-G, and Siglec-H did not (data not shown). In humans, the finding that the quantitatively major Siglec expressed by microglia, Siglec-8, and a microglial AD risk gene protein, CD33, bound to this isoform implicates the ligand in human microglial function. In mice, Siglec-F provides an interesting correlation. Siglec-F is expressed in mouse microglia but is not as abundant as mCD33 and Siglec-H. However, it is induced >26-fold in a misfolded protein (prion) mouse model of neurodegeneration (36, 37), making it a quantitatively major mouse microglial Siglec in the context of proteinopathy. Like Siglec-8, Siglec-F in peripheral tissues is expressed by eosinophils and regulates eosinophilic inflammation *via* sialoglycan binding (38, 39). Siglec-F, Siglec-8, and CD33 also have overlapping glycan-binding specificities (20, 21). These observations raise the possibility that Siglec-F and RPTPζ together regulate microglial activation levels in mouse proteinopathy, neuroinflammation, and in mouse models of human neurodegenerative diseases. This concept was strongly supported by phosphoproteomic screen that revealed upregulation of Siglec-F in three different mouse models of AD (27). The same study went on to show, using immunohistochemistry, that Siglec-8 expression is increased twofold in brain microglia of late-onset AD tissue donors compared with nondemented control donors.

The glycan-binding specificities of Siglec binding to RPTPζ^S3L^ are consistent with decoration of the large RPTPζ protein isoform with sialylated keratan sulfate proteoglycans that act as the primary Siglec-binding partners. This conclusion is based on (i) the high stringency of Siglec-8 glycan binding (19), (ii) the finding that Siglec-8 affinity capture quantitatively captured CD33 binding from human cortical extracts and Siglec-F binding from mouse cortical extracts (Figs. 4 and 9), and (iii) CD33, Siglec-8, and Siglec-F binding to extracts is reversed by treatment with sialidase and keratanase I. This conclusion is consistent with structural and thermodynamic studies of Siglec-8 binding, which demonstrate high specificity for sialylated sulfated galactose (Fig. 8*A*). The Siglec-8 glycan-binding pocket has multiple cationic subsites that match the precise spacing of a sialic acid and a sulfate attached to the same galactose (19). CD33 binding is less well defined, but recent glycan array results indicated that it shares binding to that same structure (21). Likewise, mouse Siglec-F bound robustly to this glycan motif (20). Our conclusion is that the specific biosynthetic machinery required to build terminally sialylated keratan sulfate on RPTPζ was selected in evolution to regulate microglia. Several of the glycosyltransferase and carbohydrate sulfotransferase genes required for biosynthesis of keratan sulfate are known, with some variation between peripheral and brain forms (40, 41). Our finding that *Chst1* and *St3gal4* gene products are required for binding of Siglec-F (as well as Siglec-8 and CD33) to mouse brain is consistent with their enzyme activities and distribution. These data are supported by studies of glycan biosynthetic gene expression in the human human embryonic kidney 293 cell line, in which transfection with *CHST1* is required for CD33 and Siglec-8 binding, and subsequent knockout of *ST3SIA4* reduces or eliminates binding of both Siglecs.

Another carbohydrate sulfotransferase, *Chst2*, which is required for keratan sulfate elongation, was shown to modulate Aβ deposition (42). Mice with a disrupted *Chst2* gene when crossed to J20 human amyloid precursor protein–expressing mice had increased Aβ phagocytosis and decreased Aβ deposition. These data are consistent with a functional role for keratan sulfates in microglial regulation. Further studies will be required to determine if the terminal Siglec-binding determinant (Fig. 8*A*) on keratan sulfate is the reason why.

The enzyme keratanase I, which selectively cleaves low sulfate (monosulfated disaccharides) regions of keratan sulfate, converted RPTPζ^S3L^ to a nonbinding form, whereas keratanase II, which cleaves highly sulfated keratan sulfate (disulfated disaccharides), did not. This is consistent with the finding that 98 to 99% of brain keratan sulfate is in the monosulfated disaccharide form (Galβ1–4[6-SO_4_]GlcNAcβ1–3)_n_ in mice (42). The ratio of components in human brain keratan sulfate (GlcNAc/Gal/sulfate 1:1:1) also indicate primarily monosulfated disaccharides, in which the single sulfate is biosynthetically restricted to the GlcNAc residue (43). We conclude that the terminal Siglec-engaging glycan determinant (Neu5Acα2–3[6-SO_4_]Galβ1–4[6-SO_4_]GlcNAc) is at the distal end of keratan sulfate chains with relatively low overall sulfation (monosulfated disaccharides). The detailed nature of the Siglec-binding keratan sulfate chains has yet to be determined.

RPTPζ is found as membrane-bound and membrane released (phosphacan) forms. Preliminary experiments with mouse brain indicated that about half the Siglec-F ligand is soluble in saline without detergents or chaotropic agents (data not shown), indicating that at least a portion of the Siglec ligand in mouse brain is in the phosphacan form. The histological pattern of CD33-Fc and Siglec-8-Fc binding (Fig. 10) indicates that much of the RPTPζ^S3L^ is extracellular, with a portion in structures consistent with perineuronal nets (44). RPTPζ is critical for perineuronal net structures (45), although any role for the minor RPTPζ^S3L^ isoform/glycoform has not been determined. We conclude that RPTPζ^S3L^ is distributed in the extracellular matrix of the human brain parenchyma, where it engages microglia.

A notable finding of the current study is that RPTPζ^S3L^ is expressed at greater than twofold higher levels in AD brain extracts compared with those from age-matched control donors (Fig. 5*C*), despite prior findings that mass levels of total brain keratan sulfate are reduced in AD (46). We conclude that the minor subpopulation of keratan sulfate chains bearing Siglec-reactive termini on RPTPζ is selectively upregulated in AD. Higher levels of ligands for immune inhibitory Siglecs are expected to inhibit microglial phagocytosis. Given that RPTPζ^S3L^ binds to both CD33 and Siglec-8, targeting the ligand may decrease Siglec-mediated microglial inhibition and reduce the burden of misfolded proteins. Whether disrupting RPTPζ^S3L^ reduces AD progression is a question for future studies.

## Experimental procedures

### Human microglia gene expression

Siglec expression data from human microglia were published by Alsema *et al.* (17). Bulk RNA sequencing of microglia isolated from superior parietal lobe and/or superior frontal gyrus of ten nondemented human donors was reported as log2 counts per million reads. Expression data were extracted, averaging data from different cerebral cortex areas where reported.

### Human brain tissues

Deidentified frozen human cerebral cortex samples obtained from the inferior parietal lobule of AD and age-matched nondemented donors were kindly provided by the Brain Resource Center of the Johns Hopkins Alzheimer’s Disease Research Center. Tissue blocks from across the gyrus, containing both gray and white matter, were provided. Donor information for these ten samples is shown in Table 2. An additional sample of deidentified frozen human superior frontal gyrus from a nondemented 31-year-old female donor was obtained from the same source. Formalin-fixed paraffin-embedded normal human brain cortex from a 43-year-old nondemented male donor was purchased from Amsbio.

### Mouse brains

Wildtype C57BL/6 mice were bred in house or obtained from The Jackson Laboratory. Mice with disrupted sialyltransferase genes (*St3gal1*, *St3gal2*, *St3gal3*, and *St3gal4*) were described previously (47, 48, 49, 50) and were kindly provided by Dr Jamey Marth (Sanford Bunham Prebys). Mice with a disrupted *Chst1* gene, derived as described (51), were obtained from the Knockout Mouse Project, and are available through the Mutant Mouse Resource and Research Centers (https://www.mmrrc.org). Mice with a disrupted *Ptprz1* gene were generated as described (52). Mice were deeply anesthetized, transcardially perfused with phosphate-buffered saline, brains quickly removed, sagittally bisected, and flash frozen. Brain tissue collections were performed using procedures approved by the Upstate Medical University Institutional Animal Care and Use Committee (*Ptprz1*-null mice and matched controls) or approved by the Johns Hopkins University Animal Care and Use Committee (all other mice).

### Tissue extraction and Siglec ligand purification

Brain tissues were weighed and placed in 10 ml per gram wet weight of extraction buffer (6 M GuHCl, 100 mM DTT, 5 mM EDTA, 20 mM sodium phosphate [pH 6.5], 1:100 [v/v] protease inhibitor cocktail [MilliporeSigma; catalog no.: P8340]). Tissues were homogenized ten strokes using a Potter–Elvehjem homogenizer, incubated at 4 °C for 16 h with end-over-end mixing, centrifuged at 3000*g* for 1 h, and the clear supernatant was collected.

The crude extract was dialyzed against urea buffer (1 M urea, 20 mM phosphate buffer, pH 7.4) using 100 kD molecular weight cutoff (MWCO) dialysis prior to purification. Alternatively, ligand was partially purified by differential ethanol precipitation. Crude extract was adjusted to 40% (v/v) ethanol, incubated 16 h on ice, centrifuged at 46,900*g* for 3 h, and the supernatant recovered. Additional ethanol was added to adjust the supernatant to 60% (v/v) ethanol, and then the mixture was incubated and centrifuged as aforementioned. The 60% ethanol supernatant was discarded, and the pellet was resuspended in size-exclusion buffer (4 M GuHCl and 20 mM sodium phosphate [pH 7.0]).

Buffer-exchanged or ethanol-precipitated and redissolved samples (5 ml) were loaded onto a HiPrep 26/60 Sephacryl S-500 HR size-exclusion column on an ÄKTA chromatography system (GE Healthcare) run at a flow rate of 1.0 ml/min using urea buffer or size-exclusion buffer, respectively. After injection, 48 ml of eluate were discarded, and then 1.8 ml fractions were collected until the column volume (320 ml) was eluted. Aliquots from alternate fractions were dialyzed against urea buffer for gel electrophoresis and Siglec ligand detection (see later). Fractions containing Siglec ligand were combined, the buffer was exchanged for urea buffer, and the fractions were concentrated by ultrafiltration using a 100 kD MWCO centrifugal filter.

Concentrated combined size-exclusion fractions were precleared by mixing overnight at 4 °C with 200 μl of Protein G magnetic beads (GE Healthcare) preloaded with 250 μg of human immunoglobulin G (IgG)-Fc (MilliporeSigma). The beads were removed, and the cleared supernatant was mixed at 4 °C overnight with protein G magnetic beads preloaded with 250 μg of Siglec-8-Fc. Unbound material in the supernatant was collected, and the beads were washed multiple times (0.5 ml each) with urea buffer followed by wash buffer (1 M urea, 150 mM NaCl, 20 mM sodium phosphate [pH 7.4]). The ligand was then eluted by consecutive incubations (0.25 ml each) with the same buffer containing 1 M NaCl.

### Siglec ligand electrophoresis, Siglec overlay, and immunoblotting

Siglec-Fc chimeras were produced by cloning the nucleotide sequence for the entire extracellular domain of each Siglec in frame with the human Fc domain of IgG1 behind an elongation factor 1α promoter. Chimeras were transiently expressed in human embryonic kidney 293T cells, and soluble expressed constructs were purified using Protein G chromatography (20). Alternatively, Siglec-8-Fc was produced as described previously (53).

Samples in GuHCl-containing buffers were dialyzed against urea buffer prior to electrophoresis. Proteins were resolved by SDS gel electrophoresis on composite agarose–acrylamide gels (2% agarose and 1.5% acrylamide) for 2.5 h at 100 V as described (54). Resolved proteins were electroblotted onto PVDF membranes (iBlot2; Thermo Fisher Scientific). Membranes were blocked with 5% nonfat dry milk dissolved in Dulbecco’s PBS supplemented with 0.1% Tween-20 (PBST) for 30 min. Siglec-Fc (1 μg) and horseradish peroxidase–conjugated antihuman Fc (MilliporeSigma; 0.7 μg) were incubated in a total of 50 μl of PBST for 30 min on ice and then diluted to 1 ml with PBST. Blots were overlaid with the precomplexed mixture for 16 h at 4 °C, washed, and Siglec-Fc binding was detected using enhanced chemiluminescence to reveal Siglec ligands. Images were captured using a Syngene PXi6 imaging system and quantified using ImageJ (National Institutes of Health).

For near-infrared fluorescent double labeling, blots were blocked as aforementioned and then incubated at ambient temperature with rabbit polyclonal antimouse RPTPζ (H-300; Santa Cruz; 1:1000 dilution) or rabbit polyclonal antihuman RPTPζ (PA5-53280; Invitrogen; 1:1000 dilution) in PBST containing 1% nonfat dry milk for 2 h, after which precomplexed Siglec-Fc was added (1 μg/ml of Siglec-Fc in PBST incubated for 30 min at 4 °C with 0.5 μg/ml of unconjugated goat antihuman IgG, Fc specific [catalog no.: I2136; MilliporeSigma]). After further incubation for 16 h at 4 °C, the blot was washed three times with PBST and then overlaid with PBST containing IRDye 680RD donkey anti-rabbit IgG (catalog no.: 926-68071; LI-COR Biosciences; 1:2000 dilution) to detect anti-RPTPζ and IRDye 800CW donkey antigoat IgG (catalog no.: 926-32214; LI-COR; 1:4000 dilution) to detect precomplexed Siglec-Fc. After 1 h at ambient temperature, the blots were washed with PBST and scanned with an Odyssey CLx infrared imager (LI-COR). Band intensities were quantified using LI-COR Image Studio software.

A custom molecular weight marker was prepared by mixing 1 mg/ml of human immunoglobulin M (Thermo Fisher Scientific) with 2.5 mM bis(sulfosuccinimidyl)suberate (Thermo Fisher Scientific) in PBS for 20 min, followed by addition of 90 mM Tris–HCl. The marker was stained using Visio real-time stain (Advansta), resulting in bands visible under white light or 700-nm infrared light at ∼950 kDa (pentamer) and ∼1.9 MDa (decamer).

In some experiments (Fig. 5*B*), replicate aliquots of tissue extracts were resolved on 4 to 12% polyacrylamide gels (NuPAGE Bis–Tris; Thermo Fisher Scientific) in MOPS running buffer at 120 V for 1 h, transferred to PVDF membranes, and stained with LI-COR Revert 700 total protein stain for Western blot normalization using the manufacturer’s protocols. Band intensities were quantified using LI-COR Image Studio software.

Prior to electrophoresis, some aliquots of selected samples (as indicated) were dialyzed against PBS and then treated with glycohydrolases as described previously (22). For each enzyme, control incubations were performed under identical conditions without enzyme. Enzymes (*Vibrio cholerae* sialidase, *Pseudomonas* spp keratanase I, *Bacillus circulans* keratanase II, *Proteus vulgaris* chondroitinase ABC, and *Flavobacterium meningosepticum* PNGase F) were expressed and purified or purchased as described previously (22).

### Proteomic MS

Peptides were analyzed using two protocols. The first protocol was as described previously (23). Briefly, affinity-purified ligand was desalted by ultrafiltration, reduced with DTT, and carbamidomethylated with iodoacetamide prior to digestion with Lys-C and trypsin. The resulting peptides were bulk purified using C18 Tips (Thermo Fisher Scientific) and then subjected to LC–MS using an Orbitrap Fusion Lumos tribrid mass spectrometer (Thermo Fisher Scientific) equipped with UltiMate3000 RSLCnano liquid chromatograph using a C18 analytical column. Peptides were fragmented using higher energy collisional dissociation, electron transfer dissociation, and collision-induced dissociation. Full scan mass spectra were acquired in the positive ion mode over the range *m/z* = 400 to 1600 using the Orbitrap mass analyzer in profile format with a mass resolution setting of 60,000. MS2 scans were collected in the quadrupole or ion trap for the most intense ions. Data were processed with Proteome Discoverer (version 2.4; Thermo Fisher Scientific) using UniProt UP000005640 database of 79,052 human proteins (20,361 reviewed) set to two maximum missed cleavages and static modifications of cysteine carbamidomethylation and methionine oxidation.

Alternatively, protein preparation was optimized for proteoglycans as described (55, 56). Size excluded Siglec-8 ligand was treated with PNGase F, purified by affinity chromatography, reduced with 10 mM DTT for 1 h at 37 °C and then alkylated at ambient temperature in the dark with 30 mM iodoacetamide. The sample was then dialyzed against 2 M urea, 50 mM Tris–HCl, 5 mM CaCl_2_, pH 8.0 for 1 h at ambient temperature. The dialyzed sample was proteolyzed with 300 ng/ml of endoproteinase Lys-C (catalog no.: P8109S; New England Biolabs) at ambient temperature for 5.5 h followed by addition of an equal volume of 2× modified trypsin reaction buffer (100 mM Tris–HCl, 40 mM CaCl_2_, pH 8.0) and 150 ng/ml of Trypsin-ultra Mass Spec Grade (catalog no.: P8101S; New England Biolabs). After incubation for 16 h at ambient temperature, the reaction was filtered using a 5 kDa MWCO filter (Spin-XR UF 500; Corning). The filtrate was recovered and further purified using a Water Oasis HLB SPE column (catalog no.: WAT094225) following the manufacturer’s protocol. Peptides were eluted in methanol and evaporated.

Samples were analyzed on a Q Exactive mass spectrometer with an EasyNLC 1200 nanoflow chromatography system (Thermo Fisher Scientific). Peptides were separated over a 70 min liquid chromatography (LC) gradient on an EasySpray 15 cm NanoLC column and 3 cm trapping column (both containing PepMap 100 stationary phase) at a flow rate of 300 nl/min. NanoLC buffers were 0.1% aqueous formic acid (buffer A) and 80% acetonitrile 20% buffer A (buffer B). Peptides were eluted by gradient elution (2–24% buffer B, 0–60 min; 24–36% buffer B, 60–70 min). The Q Exactive was operated in data-dependent mode with 70,000 resolution MS1 scans followed by the fragmentation of the ten most intense precursor ions at 17,500 resolution with isolation width of *m/z* 2. The peptide match function was used to preferentially select ions with isotopic distributions expected for tryptic peptides, and dynamic exclusion was used to restrict repeat fragmentation of selected ions. Thermo .RAW files were processed in Proteome Discoverer 2.4 using the default workflow for Q Exactive peptide identification. Spectra were searched against the human protein database (SwissProt TaxID = 9606; v2017-10-25) of 42,252 entries set to two maximum missed cleavages and static modifications of cysteine carbamidomethylation and methionine oxidation. In addition, the common Repository of Adventitious Proteins (cRAP) database of common laboratory contaminants (www.gpm.org) was applied using the SequestHT algorithm with a 10 ppm MS1 tolerance and a 0.02 Da MS/MS tolerance. The SequestHT identified spectra were filtered by the Percolator semisupervised learning algorithm to an estimated maximum false discovery rate of 1%. Peptide spectral matches were assembled into peptide group and protein group identifications with target decoy–based false discovery rates with a maximum target of 1% at each level using the default vendor settings for the basic consensus workflow.

### Siglec overlay histochemistry and immunohistochemistry

For immunofluorescent detection, tissue sections were deparaffinized, hydrated, and treated with 1 mM EDTA and 0.05% Tween-20 in Tris buffer pH 9 at 95 °C for 20 min for antigen retrieval. Tissues were overlaid with PBS or PBS containing 60 mU/ml sialidase (negative control) for 16 h at 37 °C, washed, then blocked at ambient temperature for 30 min with Triton–PBS (PBS, 0.1% Triton X-100) containing 30 mg/ml bovine serum albumin, followed by 10 min in enzyme blocker (BLOXALL; Vector Laboratories), and then 30 min in human Fc receptor blocker (Innovex). After blocking, slides were washed, then overlaid with Triton–PBS containing 10 mg/ml bovine serum albumin for 16 h at 4 °C containing either rabbit antihuman RPTPζ (catalog no.: PA5-53280; Invitrogen; 1:500 dilution), CD33-Fc (15 μg/ml premixed with unlabeled goat antihuman Fc 7 μg/ml), or Siglec-8-Fc precomplexed as for CD33-Fc. After incubation, slides immunostained with anti-RPTPζ were stained with Alexa Fluor Plus 488 donkey anti-rabbit antibody (catalog no.: A32790; Thermo Fisher Scientific; 1:1000 dilution), and slides were overlaid with precomplexed Siglecs with Alexa Fluor Plus 594 donkey antigoat antibody (catalog no.: A32758; Thermo Fisher Scientific; 1:500 dilution) for 2 h at ambient temperature in the dark. After incubation, slides were washed with PBS, then incubated with antiquenching reagent (catalog no.: SP-8400; Vector), followed by incubation with 4′,6-diamidino-2-phenylindole solution (10 μg/ml) for 10 min. The slides were washed with PBS and mounted with ProLong Diamond Antifade Mountant (Thermo Fisher Scientific). Slides were imaged using a Nikon Eclipse 90i microscope (Nikon Instruments).

## Data availability

Proteomic MS data are available at JPOST (https://repository.jpostdb.org/entry/JPST001519.0) for the Orbitrap data and at FigShare (https://dx.doi.org/10.6084/m9.figshare.19319588) for the Q Exactive data. Other source data will be shared upon request to Ronald Schnaar (schnaar@jhu.edu).

## Supporting information

This article contains supporting information.

## Conflict of interest

The authors declare that they have no conflicts of interest with the contents of this article.
