# Supplementary material for: Human brain sialoglycan ligand for CD33, a microglial inhibitory Siglec implicated in Alzheimer’s disease
Source: J Biol Chem. 2022 Apr 20;298(6):101960. doi: 10.1016/j.jbc.2022.101960 (PMC9130525; doi:10.1016/j.jbc.2022.101960)
Supplement: Supplemental Figures S1–S3 and Table S1 [file mmc1.pdf]

Supporting Information

Human brain sialoglycan ligand for CD33, a microglial inhibitory Siglec implicated in Alzheimer's disease

Anabel Gonzalez-Gil, Ryan N. Porell, Steve M. Fernandes, Eila Maenpaa, T. August Li, Tong Li, Philip C. Wong, Kazuhiro Aoki, Michael Tiemeyer, Zaikuan J. Yu, Benjamin C. Orsburn, Namandjé N. Bumpus, Russell T. Matthews, and Ronald L. Schnaar

Contents:

Table S1 – RPTPζ peptides found in purified RPTPζ^{S3L} by proteomic mass spectrometry.
Figure S1 – Keratanase I or sialidase treatment of purified RPTPζ^{S3L} eliminates Siglec-Fc binding without loss of RPTPζ immunoreactive protein.
Figure S2 – Expression of RPTPζ in control (non-demented) and AD cerebral cortex extracts.
Figure S3 – Binding of microglial inhibitory Siglecs to human cerebral cortex extracts.

Table S1 – RPTPζ peptides found in purified RPTPζ^{S3L} by proteomic mass spectrometry. Purified RPTPζ^{S3L} was subjected to proteomic mass spectrometry in two different MS systems as indicated.

System	Sequence	Modifications	# Missed Cleavages	Theo. MH+ [Da]	Charge	m/z [Da] Sequest HT	XCorr Sequest HT	Amanda Score
Orbitrap	VSGGVSEMVFK	1×Oxidation [M8]	0	1155.57151	2	578.29474	3.54	
Orbitrap	FAVLYQQLDGEDQTK		0	1754.85962	2	877.94165	4.51	
Orbitrap	DIEEGAIVNPGR		0	1269.64343	2	635.33179	4.19	
Q Exactive	VSGGVSEMVFK	1×Oxidation [M8]	0	1155.57144	2	578.28763		268.07
Q Exactive	CMSCSSYR	2×Carbamidomethyl [C1; C4]	0	1050.38015	2	525.69724		185.27
Q Exactive	AIIDGVESVSR		0	1145.61608	2	573.31323		268.16

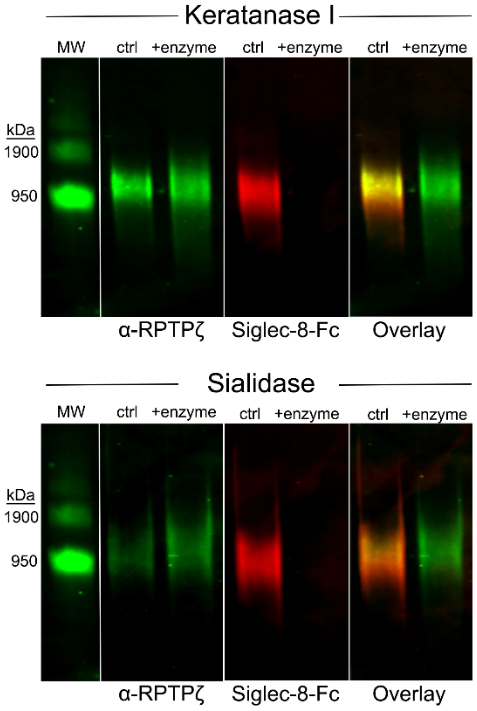

Figure S1. Keratanase I or sialidase treatment of purified RPTPζ^{S3L} eliminates Siglec-Fc binding without loss of RPTPζ immunoreactive protein. RPTPζ^{S3L} purified from human brain was treated with keratanase I (8.4 mU/ml, 37°C, 18 h), sialidase (152 mU/ml, 37°C, 1.5 h) or under the same conditions without enzyme (ctrl). At the end of the enzymatic treatments, proteins were electrophoretically resolved on composite agarose-acrylamide gels, transferred to PVDF membranes, and double-probed with anti-RPTPζ (α-RPTPζ, green) and with Siglec-8-Fc (red). The results indicate that Siglec-8-Fc binding was abrogated whereas RPTPζ immunoreactive protein remained.

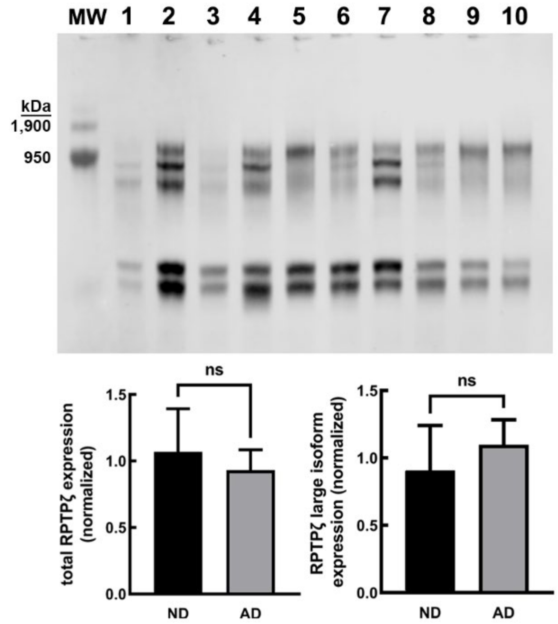

Figure S2. Expression of RPTPζ in control (non-demented) and AD cerebral cortex extracts. Proteins were extracted and resolved on replicate composite agarose-acrylamide gels to resolve large proteins. Proteins were blotted to PVDF membranes and the blots double near-infrared labeled for Siglec ligands (CD33-Fc and Siglec-8-Fc overlay, main text) and with rabbit polyclonal anti-human RPTPζ detected with IRDye 680RD donkey anti-rabbit IgG (LI-COR, top panel). Since anti-RPTPζ blot images for the replicate blots were comparable the anti-RPTPζ blot image for the CD33-Fc double labeled blot is shown (left) with molecular weight markers of prelabeled crosslinked IgM. Lanes 1-5, control donor samples; Lanes 6-10, AD donor samples.

For normalization, equal aliquots from each donor were subjected to 4-12% acrylamide gel electrophoresis to resolve total extracted proteins and blots were stained with LI-COR Revert 700 total protein stain (main text). Total RPTPζ band densities and the largest RPTPζ isoform band density were determined, normalized to total protein, and aggregate data for control (non-demented) compared to AD donor samples are shown.

Figure S3. Human cortical extracts probed with different human microglia-expressed inhibitory Siglecs.

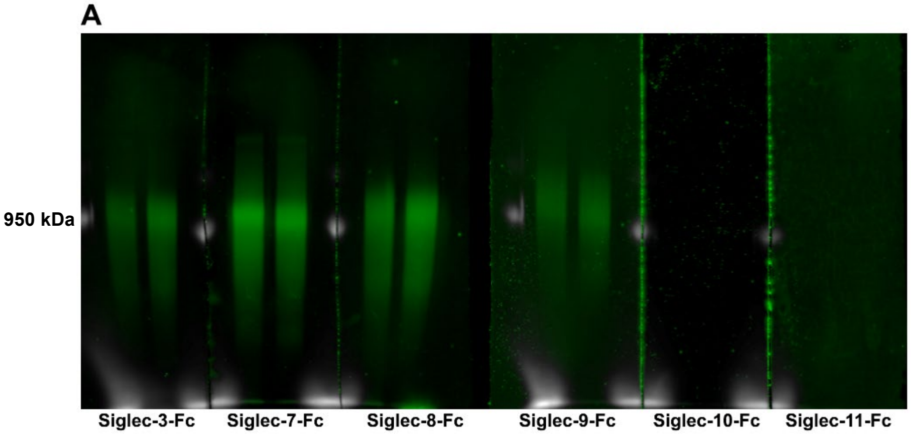

A, Aliquots of combined extract from multiple control (non-demented) human cerebral cortices were resolved in duplicate lanes flanked by prelabeled crosslinked IgM molecular weight marker. Blots were cut at or near the IgM markers and pieces separately incubated with the indicated Siglec-Fc chimera pre-complexed with goat anti-human Fc and IRDye 800CW donkey anti-goat IgG. After washing, blots were aligned and infrared images captured together. Siglec binding (800 nm) is represented in green and molecular weight standards (700 nm, 950 kDa) in grey.

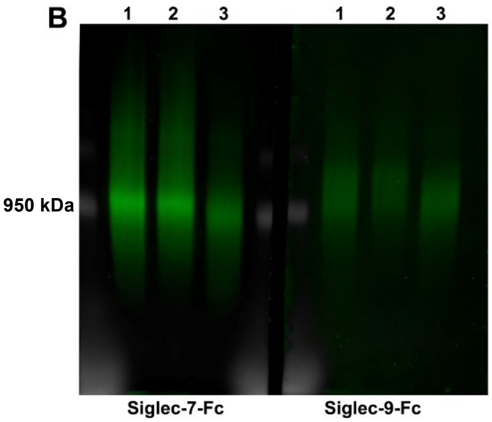

B, Siglec-8 ligand extracted from control human cerebral cortices was partially purified by size exclusion chromatography as in Fig. 3A. Partially purified extract was subjected to Siglec-8-Fc affinity capture as in Fig. 3B. Replicate aliquots of pre-cleared sample (lane 1), unbound material (lane 2) and salt eluted sample (lane 3) were resolved flanked by crosslinked IgM molecular weight marker. Resolved proteins were transferred to PVDF membrane. The membrane was cut and sections separately incubated with the indicated precomplexed Siglec-Fc chimera and binding determined as described for panel A above. Siglec binding (800 nm) is represented in green and molecular weight standards (700 nm, 950 kDa) in grey.
